# Evaluation of Facial Artery Musculomucosal Flap for Reconstruction of Small Tongue Defects

**DOI:** 10.1055/s-0045-1809954

**Published:** 2025-10-29

**Authors:** Shruti Kongara, Kishore Purushothaman, Jimmy Mathew, Yogesh Dhoke, Arya Chandrababu Jaya, Srilekha Reddy Galigutta, Shravan Rai, Abhinandan Badam, Krishnakumar Thankappan, Subramania Iyer

**Affiliations:** 1 Department of Plastic and Reconstructive Surgery, Amrita Institute of Medical Sciences, Kochi, Kerala, India; 2 Department of Head and Neck Surgery, Mazumdar Shaw Medical Centre, Narayana Hospital, Bangalore, Karnataka, India; 3 Department of Head and Neck Surgey and Oncology, Amrita Swallow Centre, Amrita Institute of Medical Sciences, Kochi, Kerala, India; 4 Department of Plastic and Reconstructive Surgery, Amrita Hospital, Faridabad, Haryana, India; 5 Department of Plastic and Reconstructive Surgery, Broomfield Hospital, Chelmsford, United Kingdom; 6 Department of Head and Neck Surgery and Oncology, Amrita Institute of Medical Sciences, Kochi, Kerala, India

**Keywords:** tongue, glossectomy, speech intelligibility, facial artery musculomucosal flap

## Abstract

**Background:**

Partial glossectomy defects can be managed by different methods, ranging from primary closure to pedicled or free flaps. The facial artery musculomucosal (FAMM) flap provides an excellent match to lingual tissue and provides an inconspicuous donor site. This study aims to compare functional outcomes, especially speech, of patients with partial glossectomy defects (≤1/3rd of tongue) reconstructed by FAMM flap with those of similar defects closed primarily or healed by secondary intention. It also offers to resolve the question of whether defects of this size should be reconstructed at all.

**Materials and Methods:**

A total of 25 patients with T1 or T2 oral tongue cancer undergoing resection and reconstruction with islanded FAMM Flap were included in the prospective limb of the study. Retrospective comparison was done with patients of similar defects who had primary closure (25 patients) or were allowed healing by secondary intention (25 patients). Their peri-operative parameters and functional outcomes were compared.

**Results:**

The FAMM flap group required longer duration of nasogastric feeds and overall hospital stay. Speech intelligibility, as assessed by a speech therapist after 3 months, was satisfactory in all the groups of patients. Results of subjective assessment of speech-related problems did not demonstrate any advantage to the flap group.

**Conclusion:**

Patients with small tongue defects, irrespective of method of repair, have good speech outcomes. There was no significant difference between flap and non-flap groups in objective speech intelligibility outcomes. The benefit of reconstructing defects less than or equal to one-third of the tongue is questionable.

## Introduction

Many methods are in use for reconstruction of glossectomy defects, ranging from primary closure to local, regional, and free flaps. 1 Small defects can be closed primarily or left for healing by secondary intention. Defects which are approximately one-third of the tongue are sometimes too large for primary closure, but too small for regional flaps. 2 The key is to replace adequate amount of tissue, while ensuring pliability. Myomucosal flaps from inside the cheek have thin, mobile, well-vascularized, and sensitive tissue. The facial artery musculomucosal (FAMM) flap is one such axial flap with a wide arc of rotation and relative ease of elevation. The objective of this study is to present our experience with FAMM flap for reconstruction of partial glossectomy defects (≤1/3rd of tongue) and compare it with hitherto standard methods of primary closure and healing by secondary intention. We aim to determine whether reconstructing tongue defects of this size is advantageous or not.

## Materials and Methods

**Fig. 1:**
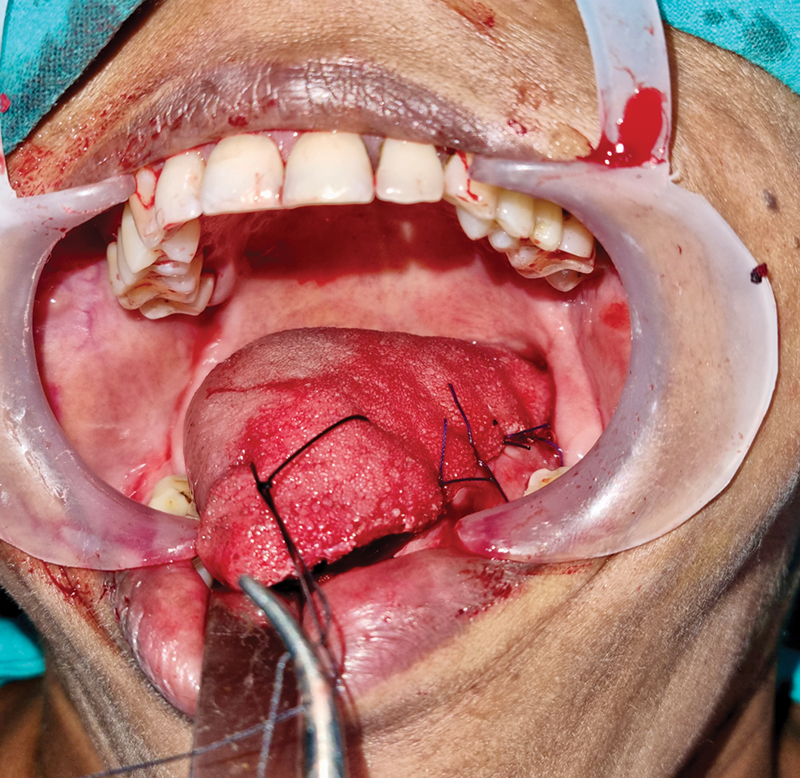
Primary closure of partial glossectomy wound.

**Fig. 2:**
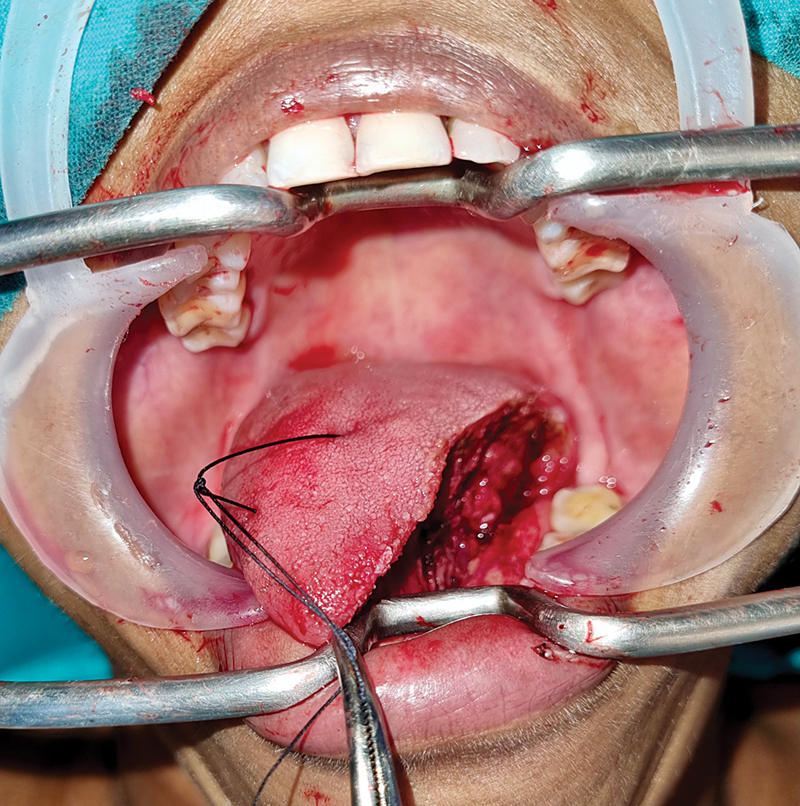
Partial glossectomy wound for healing by secondary intention.

Patients with primary oral tongue carcinoma stage T1 or T2 treated at our tertiary care hospital, in the Head and Neck Oncology and Plastic Surgery departments with upfront surgery in the form of partial glossectomy and reconstruction with FAMM flap, during the period January 2019 to June 2021, who were fluent in Malayalam language and consented to the study were recruited in the prospective limb of the study ( *n* = 25). The retrospective groups comprised 25 patients who underwent primary closure ( Fig. 1 ) and 25 patients who had healing by secondary intention ( Fig. 2 ) for similar tongue defects earlier, as was the standard of care for such defects. Their peri-operative and functional outcomes were assessed. Speech assessment was done at minimum 3 months of follow-up by the same speech therapist for all the patients. Detailed speech analysis was done for 20 patients of the FAMM flap group, who consented for the speech evaluation. Data of speech analysis were available for 10 patients of the primary closure group and 8 patients of secondary healing group, together taken as “non-flap” group ( *n* = 18) for the sake of comparison with the “FAMM flap” group.

### Surgical Technique—Islanded FAMM Flap

**Fig. 3:**
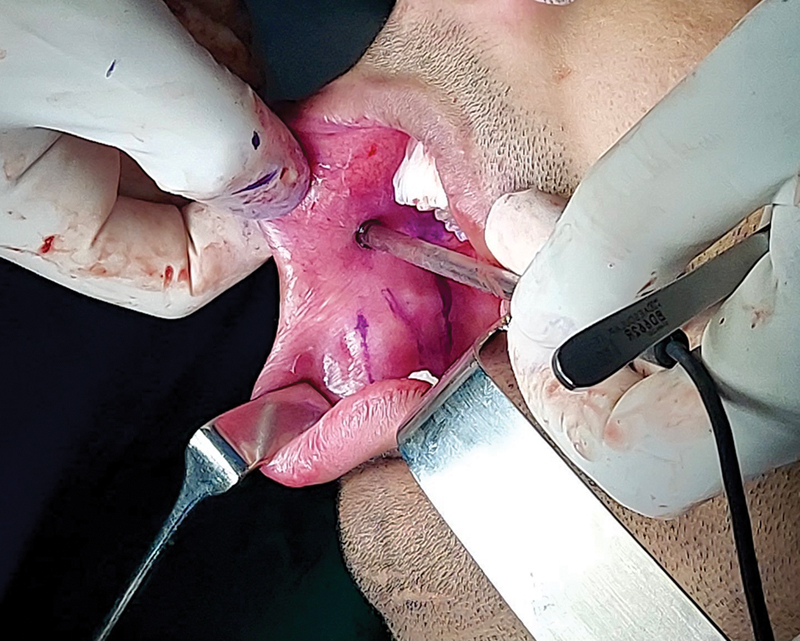
Marking of facial artery with the help of Doppler.

**Fig. 4:**
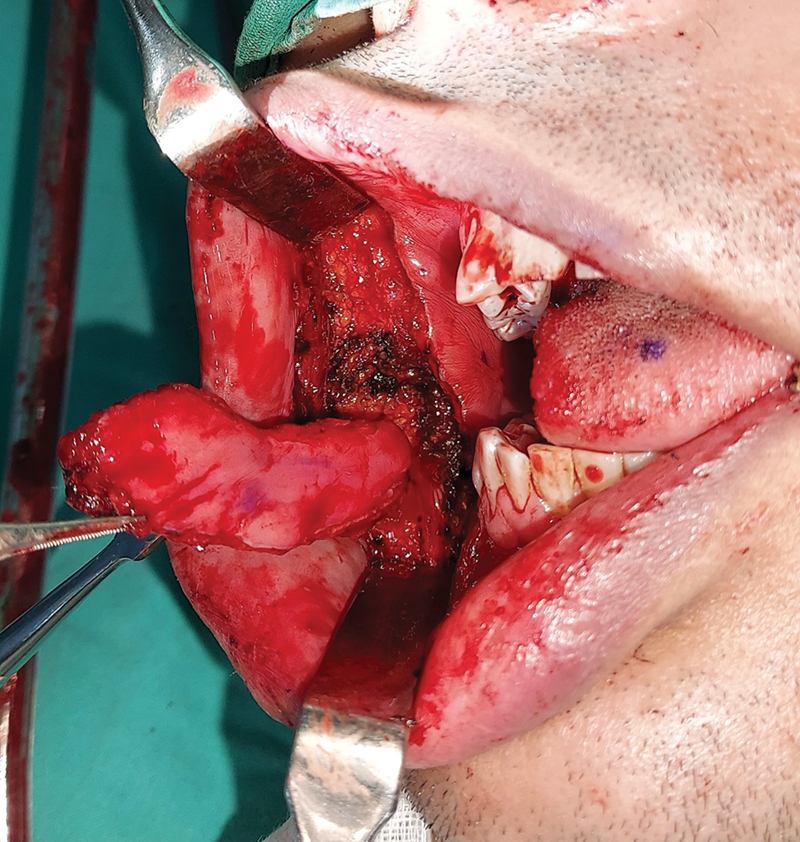
Harvested facial artery musculomucosal (FAMM) flap.

**Fig. 5:**
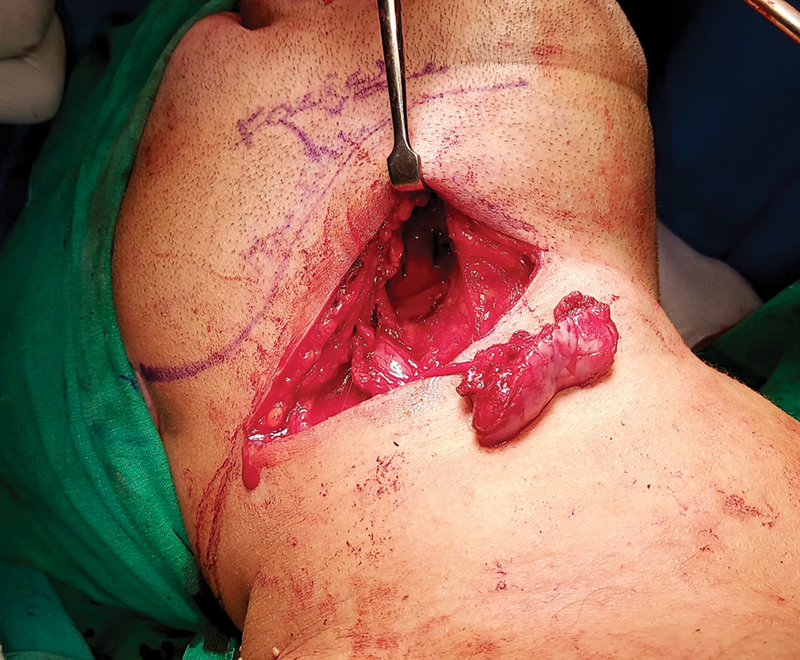
FAMM flap delivered into the neck. FAMM, facial artery musculomucosal.

**Fig. 6:**
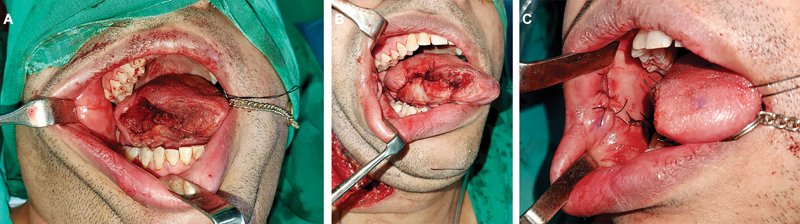
( **A** ) Partial glossectomy defect. ( **B** ) FAMM flap inset into the defect. ( **C** ) Primary closure of donor site. FAMM, facial artery musculomucosal.

**Fig. 7:**
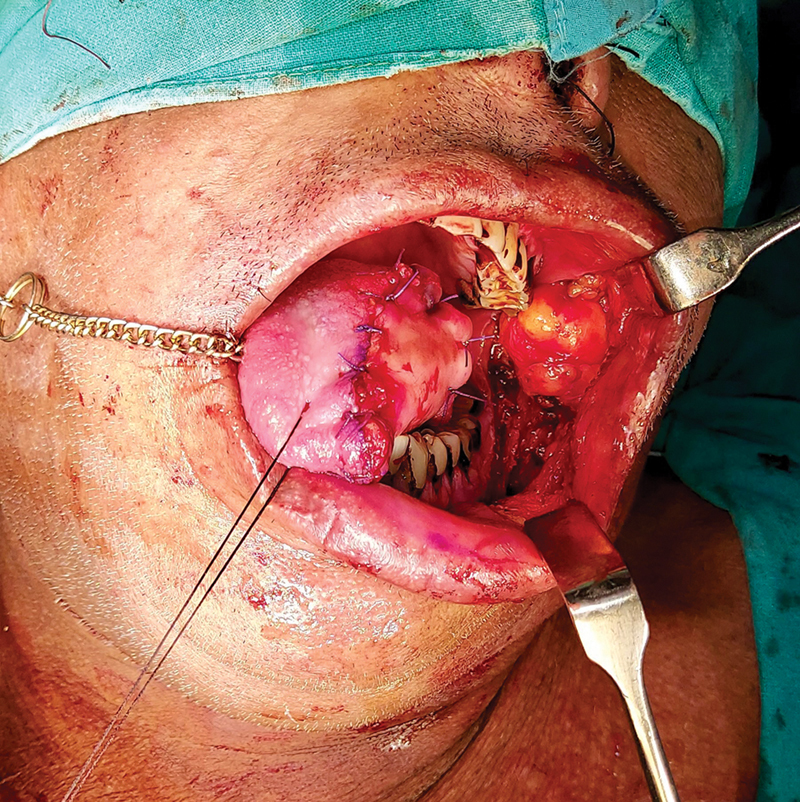
FAMM flap after inset and donor site covered by buccal fat pad. FAMM, facial artery musculomucosal.

**Fig. 8:**
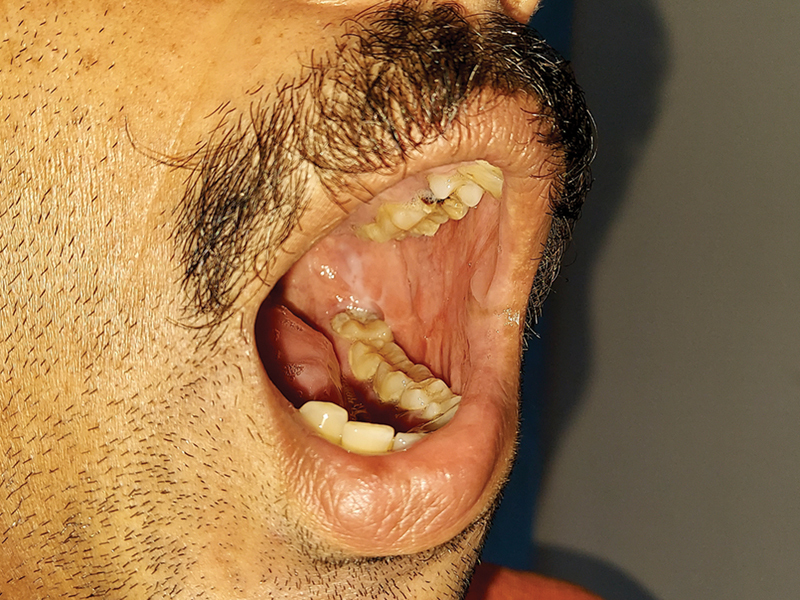
FAMM flap donor site, 6 months after surgery. FAMM, facial artery musculomucosal.

**Fig. 9:**
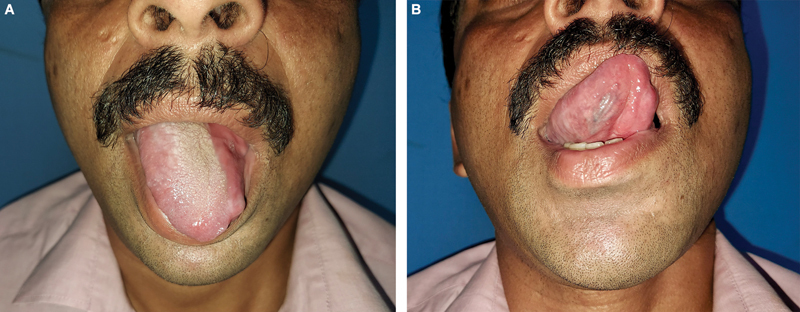
( **A, B** ) FAMM flap for partial tongue defect, 6 months after surgery.

Flap was outlined on healthy buccal mucosa, after marking the facial artery with a Doppler ( Fig. 3 ). Superior and inferior limits were 5 mm from upper and lower gingivobuccal sulcus respectively, anteriorly 1 cm from oral commissure, and posteriorly limited by the orifice of Stensen's duct. Mucosal incision was deepened through the buccinator, and plane of dissection deep to buccinator, preserving and including the facial artery. Facial vessels were ligated above the superior margin of flap. Posteriorly, the buccinator was separated from masseter to expose buccal fat pad. Inferiorly flap was separated from alveolar margins of teeth ( Fig. 4 ). Lower border of mandible was approached from the neck incision. Marginal mandibular nerve and facial vessels identified and preserved. Intra-oral flap was connected to extra-oral part after dissection along the facial pedicle ( Fig. 5 ). Flap was mobilized and taken into the neck, pedicled on the facial vessels, and carefully tunneled through the floor of mouth into the oral cavity to reconstruct the tongue defect ( Fig. 6A–C ). The donor site was either closed primarily or layered with buccal fat pad and left to mucosalize ( Figs. 7 , 8 and 9A, B ).

*Technique of primary closure* : single layered closure with 2–0 Vicryl by mucosal opposition horizontally with least disturbance of residual muscle fibers of the tongue and rotation of tongue tip.

Outcome measures which were assessed:

- Peri-operative outcomes: duration of hospitalization, duration of nasogastric feeds, escalation of pain medication, and complications.
- Functional outcomes (speech): objective assessment using Malayalam speech articulation scoring system ASIAT (Amrita Speech Intelligibility Assessment Tool) and AJYNIHH speech intelligibility rating scale. 9 Subjective speech perception and speech-related quality of life using Hirose speech score and Speech Handicap Index (SHI). 10
- Functional outcomes (swallowing): day of start of oral blend diet, scoring by Eating Assessment Tool (EAT) and Functional Oral Intake Scale (FOIS). 11 12

Statistical analysis was done using SPSS version 23.0 (Armonk, New York, United States: IBM Corp.). Categorical variables were expressed as frequencies and percentages and continuous variables as mean and standard deviation or median and range as appropriate. To test the statistical significance of differences in mean values of measurable variables among the three groups, analysis of variance was done and to test differences in percentages of categorical variables, the chi-square test was done. In case of statistical significance, the Bonferroni multiple comparison test was done to identify statistically significant pairs of groups. “ *p* ”-Value <0.05 was considered statistically significant.

## Results

The cohort was predominantly male (75%), mean age being 58.2 years (range 25–78 years). All the FAMM flap group patients ( *n* = 25) underwent neck dissection, 7 of them had adjuvant radiation. The most common site of lesion was lateral border of tongue, in 62 patients (83%). The largest defect was 60 mm × 40 mm in both the flap and non-flap groups ( Table 1 ). All of them were defects of less than or equal to one-third of the tongue.

**Table 1:** Demographics and clinical characteristics of partial glossectomy wounds managed by FAMM flap, primary closure, or secondary healing

Variables	Category	Frequency (total *n* = 75)	FAMM flap	Primary closure	Secondary healing	*p* -Value
Age	Mean	58.24	54.04	60.04	60.64	0.081
	SD	± 11.56	± 11.17	± 12.83	± 9.73	
	Range (y)	25–78	33–75	25–78	41–78	
Sex	Male	56	19 (76%)	19 (76%)	18 (72%)	0.932
	Female	19	6 (24%)	6 (24%)	7 (28%)	
T stage	T0	9	0 (0%)	5 (20%)	4 (16%)	0.071
	T1	35	10 (40%)	15 (60%)	10 (40%)	
	T2	26	15 (60%)	5 (20%)	11 (44%)	
Neck dissection	No	5	0 (0%)	3 (12%)	2 (8%)	0.223
	Yes	70	25 (100%)	22 (88%)	23 (92%)	
Adjuvant radiation	No	68	18 (72%)	25 (100%)	25 (100%)	
	Yes	7	7 (28%)	0 (0%)	0 (0%)	
Site of lesion	Tip	3	2 (8%)	1 (4%)	0 (0%)	0.082
	Right lateral border	36	9 (36%)	10 (40%)	17 (68%)	
	Left lateral border	26	9 (36%)	9 (36%)	8 (32%)	
	Dorsal tongue	2	0 (0%)	2 (8%)	0 (0%)	
	Ventral tongue	8	5 (20%)	3 (12%)	0 (0%)	
Site of defect	Tip	1	0 (0%)	1 (4%)	0 (0%)	0.117
	Anterior third	9	5 (20%)	2 (8%)	2 (8%)	
	Middle third	41	10 (40%)	15 (60%)	16 (64%)	
	Posterior third	18	5 (20%)	6 (24%)	7 (28%)	
	Ventral	6	5 (20%)	1 (4%)	0 (0%)	

Abbreviation: FAMM, facial artery musculomucosal.

The mean hospital stay of the FAMM flap group was 9 days, which was significantly more than primary closure and secondary healing groups (5.36 and 4.92 days, respectively). One FAMM flap patient stayed longer (17 days) due to peri-operative cardiac complications, resulting in a longer mean stay in the flap group. Nasogastric feeds were required for a mean of 5.7 days in the FAMM flap group, which was more than primary and secondary healing groups, 3.2 and 3.7 days, respectively ( Table 2 ). Marginal mandibular palsy was observed in eight patients in the FAMM flap group and all of them eventually recovered. Flap congestion occurred in one patient and marginal flap necrosis in one patient. In the non-flap groups, two patients had secondary hemorrhage and two had neck infection ( Table 3 ). The size of the largest flap harvested was 55 mm × 40 mm.

**Table 2:** Comparison of peri-operative outcomes of partial glossectomy wounds managed by FAMM flap, primary closure, or secondary healing

Variables	Category	Frequency (total *n* = 75)	FAMM flap	Primary closure	Secondary healing	*p-* Value
Need for escalation of analgesics	Yes	9	5 (20%)	2 (8%)	2 (8%)	0.321
	No	66	20 (80%)	23 (92%)	23 (92%)	
Duration of hospital stay	Mean	6.43	9	5.36	4.92	0.00
	SD	± 2.61	± 2.33	± 1.73	± 1.44	
	Range	2–17	6–17	2–9	3–8	
Duration of Ryles feeds	Mean	4.19	5.68	3.16	3.72	0.00
	SD	± 2.08	± 1.70	± 1.31	± 2.25	
	Range	1–13	4–10	1–7	1–13	
Post-op day of start of oral blend diet	Mean	4.77	5.44	4.16	4.72	0.008
	SD	± 1.78	± 1.47	± 1.31	± 2.25	
	Range	2–14	3–8	2–8	2–14	

Abbreviations: FAMM, facial artery musculomucosal; SD, standard deviation.

**Table 3:** Immediate and late complications in the three groups

Complications	FAMM flap ( *n* = 25)	Primary ( *n* = 25)	Secondary ( *n* = 25)
Flap congestion	1	–	–
Flap necrosis (partial)	1	–	–
Marginal palsy (recovered)	8	–	–
Neck hematoma	0	1	1
Neck infection	0	2	0
Secondary hemorrhage	0	0	2
Unrelated to surgery	1	2	0

Abbreviation: FAMM, facial artery musculomucosal.

**Fig. 10:**
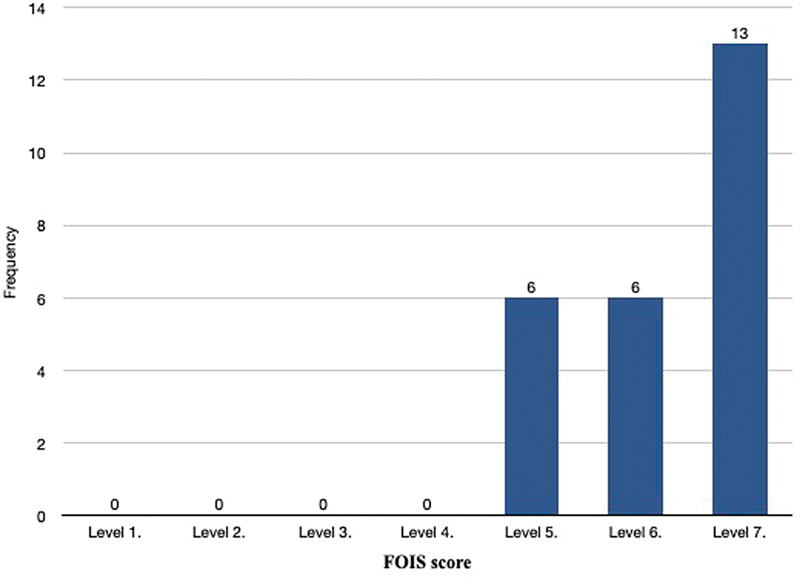
Functional Oral Intake Scale (FOIS) in FAMM Flap group. FAMM, facial artery musculomucosal.

In FAMM flap group, subjective assessment of swallowing was done by FOIS and EAT 10. Mean EAT score was 10.48, indicating “some eating abnormality.” The mean FOIS score was 6.28, with 65% of patients having level 7 “total oral intake with no restrictions” and 30% level 6 ( Fig. 10 ).

SHI indicates the patient's perception of their speech problem. All the patients had scores below 30, implying that partial glossectomy may not affect speech considerably. The “non-flap” group did better (mean score: 1.6) compared with the FAMM flap group (mean: 8.08). Hirose score is a speech ability score as perceived by the patient's family. Both groups of patients had “excellent speech ability” with a mean of 8.36 in the FAMM flap group and 9.33 for the non-flap group. There was a statistically significant difference between the groups, with regards to SHI and Hirose scores. The non-flap group had better perception of speech ability and fewer speech-related problems compared with the FAMM flap group ( Table 4 ).

**Table 4:** Comparison of Hirose and SHI scores between the flap and non-flap groups

Variable	Score (min–max)		FAMM flap group, *n* = 20	Non-flap group (primary + secondary), *n* = 18	*p-* Value
Hirose	2–10	Mean	8.36	9.33	**0.01**
		SD	± 1.15	± 1.19	
		Range	5–10	6–10	
SHI	0–120	Mean	8.08	1.61	**0.00**
		SD	± 6.56	± 2.93	
		Range	1–26	0–9	

Abbreviations: FAMM, facial artery musculomucosal; SD, standard deviation.

**Fig. 11:**
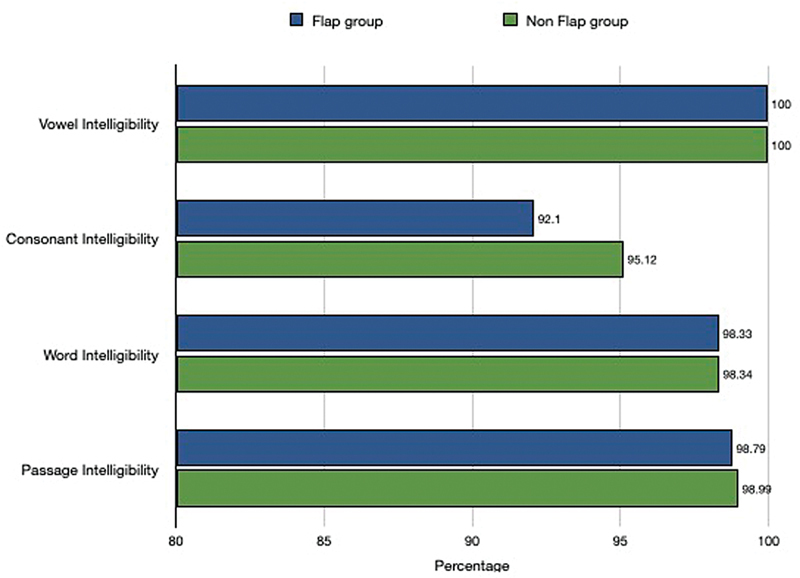
Intelligibility scores of speech parameters in the speech articulation scoring tool ASIAT (Malayalam language). ASIAT, Amrita Speech Intelligibility Assessment Tool.

There was no difference in the intelligibility of vowels, consonants, or words between the groups. The intelligibility of Malayalam passage was also comparable. The mean values in both the groups indicate that speech has “good intelligibility” after partial glossectomy. ( Fig. 11 ).

**Fig. 12:**
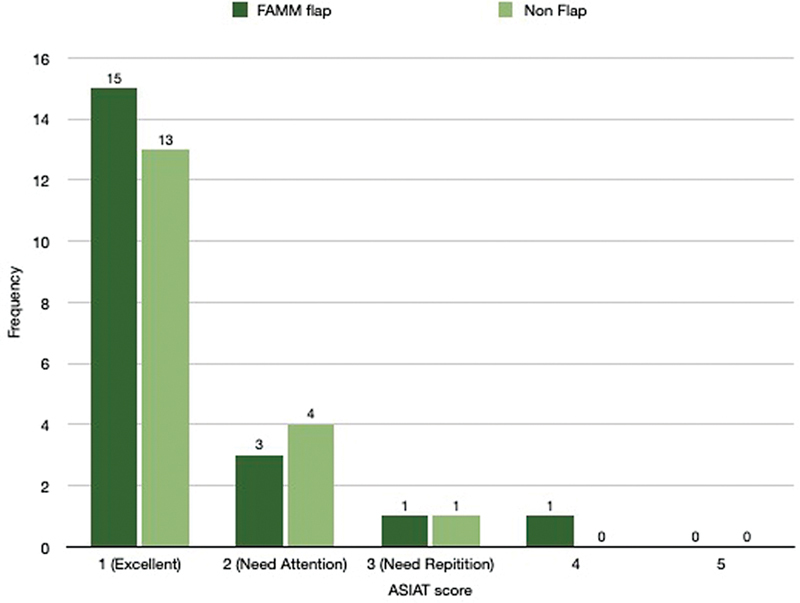
Comparative analysis of ASIAT score between the flap and non-flap groups. ASIAT, Amrita Speech Intelligibility Assessment Tool.

Overall speech intelligibility by ASIAT score was comparable in both the flap and non-flap groups ( *p* -value: 0.77). In total, 75% of the flap group and 72% of the non-flap group had “excellent intelligibility (Score 1)” ( Fig. 12 ). There was no statistically significant difference in both groups regarding intelligibility scoring by Ali Yavar Jung National Institute of Hearing Handicapped (AYJNIHH; Table 5 ). Both ASIAT and AYJNIHH scoring were done by the speech pathologist based on the intelligibility of the patient's recorded speech.

**Table 5:** Comparison of AYJNIHH score between the FAMM flap group and non-flap group

AYJNIHH score	Frequency (total *n* = 38)	FAMM flap group	Non-flap group	*p-* Value
Excellent	35 (92.1%)	18 (90%)	17 (94.4%)	1.0
Careful listening	3 (7.9%)	2 (10%)	1 (5.6%)	

Abbreviations: AYJNIHH, Ali Yavar Jung National Institute of Hearing Handicapped; FAMM, facial artery musculomucosal.

## Discussion

The aim of reconstructing the oral tongue is to ensure maximum function of the residual tongue tissue, since its complex function cannot be replicated with any reconstructive technique. 3 Traditionally, defects less than 25% of the tongue were not necessarily reconstructed. These defects, especially in the lateral tongue, were closed primarily or left to heal by secondary intention with minimal functional deficits. 4 Larger defects benefitted from pedicled or free-flap reconstruction. Skin-lined flaps are widely used but they have drawbacks like donor-site morbidity and hair growth in oral cavity. 5 Moderate-sized defects that are too large for primary closure and too small for flap reconstruction pose a challenge. 2

The facial artery myomucosal flap was introduced for small mucosal defects of the oral cavity and neck region. 6 The original pedicled flap was proposed by Pribaz et al 7 but it required a second stage to divide the pedicle. Islanding and using it in a single-stage procedure was a modification proposed by Zhao et al. 8

In our center, prior to this study, partial glossectomy defects ≤1/3rd of tongue were managed by primary closure if they did not involve floor of mouth and by secondary intention healing if floor is involved, as it was felt that primary closure may restrict tongue mobility by tethering tongue to the floor of mouth and re-orientation of muscle fibers with scarring may deter movement of residual tongue. Secondary healing would form a more pliable scar and cause lesser distortion of tongue tip.

In our study, on comparing primary closure with secondary intention healing (for similar size defects), there was no difference in the time taken to start oral feeds, duration of hospitalization, or requirement of additional opioids for pain relief. FAMM flap patients had a comparable defect size with respect to the size of native tongue, with resection remaining ≤1/3rd of tongue. The FAMM flap group had a longer duration of hospital stay and required more days of nasogastric feeds compared with the non-flap groups.

A total of 20 patients in the FAMM flap group and 18 patients in the non-flap group (10 primary closure + 8 secondary intention) underwent detailed speech analysis. Using the Malayalam speech articulation scoring system ASIAT, 9 the mean vowel, consonant, word, and paragraph intelligibility scores were found to be good and showed no significant difference between the groups, indicating good intelligibility outcomes after partial glossectomy. Lee et al 10 on studying swallowing and articulation in patients with partial glossectomy defects without reconstruction concluded that they demonstrate good function on long-term follow-up and that resection volume, age, and follow-up duration affected the relative functional recovery. 13

Many earlier reports document noticeable speech dysfunction following partial glossectomy, but most of them reported results for larger tumors and involving patients who received adjuvant radiation or chemoradiotherapy. 14 Shin et al 15 reported that radiotherapy had a detrimental effect on postoperative functional outcome following partial glossectomy with free-flap reconstruction. Radiation therapy may induce late complications such as fibrosis, mucosal edema, trismus, and salivary gland atrophy, which may have an adverse impact on swallowing and articulation. 15 Chuanjun et al 16 on comparing speech intelligibility scores between a group of patients whose tongue defects were reconstructed immediately with free radial forearm flap or pedicled flaps and another group whose tongue defects were closed primarily without reconstruction found that articulation intelligibility of the group without reconstruction was significantly higher.

Zhao et al 17 in a study of partial glossectomy defects reconstructed with an islanded FAMM flap found that the function of neo-tongue in terms of speech and swallowing was satisfactory, as the residual tongue function remained intact. Donor sites were closed primarily, and mastication and intake were not impaired after wound healing. The results are comparable to those of our FAMM flap group. Ayad et al 18 published a series of 57 oral cancer patients, who underwent floor of mouth and ventral tongue reconstruction with FAMM flap to optimize postoperative oral function and prevent tongue tethering. Tongue mobility was termed “satisfying” for 87% of patients. Speech was deemed as functional/understandable in 93% of the patients. The results are comparable to our study.

Benjamin et al 19 in their study of 21 patients with T1 and T2 oral tongue cancers who had FAMM flap reconstruction of their partial glossectomy defects found all the patients to have intelligible speech, good tongue mobility, no difficulty with mastication, and good swallowing ability. Joseph et al 5 studied 40 patients with carcinoma of the oral tongue, of which 20 underwent reconstruction with islanded FAMM flap and 20 with fasciocutaneous free flaps (FCFFs). Assessment showed excellent speech in 85% of their islanded FAMM patients. They found that islanded FAMM was as useful as FCFF for moderate-sized defects of oral tongue, especially in the elderly and those with co-morbid conditions. Regarding size-matched lateral oral tongue defects, both islanded FAMM and FCFF showed similar speech and swallowing function.

Joseph et al 20 in their study of 75 patients who underwent reconstruction of various head and neck mucosal defects by an islanded FAMM or facial artery osseomyomucosal flap found that reduction in mouth opening was associated with adjuvant radiation. They concluded that islanded FAMM seemed a better alternative to FCFFs for floor of mouth and ventral tongue defects, showing positive functional results in terms of speech and swallowing. They mentioned that marginal mandibular palsy rates following FAMM were higher and that neck dissection is known to cause marginal mandibular paresis, but FAMM flap delivery medial to the marginal mandibular nerve probably adds to it. 21 A small proportion of flap patients in our study (4 of the 20 FAMM flap patients) had adjuvant radiation, but none of the non-flap group underwent radiation. We have not separately evaluated their function, and further studies comparing patients with and without adjuvant radiation would be able to shed more light regarding the effects of radiation.

In this study, we evaluated the outcomes of using the FAMM flap for reconstruction of partial glossectomy defects of up to one-third of the oral tongue. Our findings demonstrate that while reconstruction with a FAMM flap is technically suitable and achieves good speech and swallowing outcomes, the functional results are comparable to those achieved by primary closure or healing by secondary intention for similar-sized defects. Notably, the FAMM flap group exhibited longer hospital stays and longer duration of nasogastric feeds. Objective speech intelligibility was comparably high across all the groups, although subjective patient-reported speech outcomes were slightly better in the non-flap group. Given the increased peri-operative morbidity associated with flap reconstruction and the comparable functional outcomes, the necessity of using a flap for defects ≤1/3rd of the tongue remains debatable. Further larger prospective studies are needed, especially considering the effects of adjuvant therapy, to refine the indications for FAMM flap reconstruction in small tongue defects.
